# Aspects épidémiologiques et cliniques des cancers du col de l’utérus au Cameroun: expérience de l’Hôpital Général de Douala

**DOI:** 10.11604/pamj.2022.42.109.30704

**Published:** 2022-06-09

**Authors:** Berthe Sabine Esson Mapoko, Anne Marthe Maison Mayeh, Ruth Rosine Mekah Mapenya, Esther Dina Bell Mbassi, Etienne Atenguena Okobalemba, Anne Juliette Flora Sango, Sidonie Ananga Noa, Ambroise Ntama, Zacharie Sando, Paul Ndom, Martin Essomba Biwole, Albert Mouelle Sone

**Affiliations:** 1 Faculté de Médecine et des Sciences Biomédicales, Université de Yaoundé I, Yaoundé, Cameroun,; 2 Faculté de Médecine et des Sciences Pharmaceutiques, Université de Douala, Douala, Cameroun,; 3 Faculty of Health Sciences, University of Buea, Buea, Cameroun

**Keywords:** Cancer du col de l’utérus, épidémiologie, clinique, Hôpital Général de Douala, Cameroun, Cervical cancer, epidemiology, clinical, Douala General Hospital, Cameroon

## Abstract

**Introduction:**

le cancer du col de l´utérus est un problème de santé publique au Cameroun, avec le 2^e^ rang en termes d´incidence. Notre étude avait pour objectif de décrire les caractéristiques épidémiologiques et cliniques des patientes avec cancer du col de l´utérus à l´Hôpital Général de Douala, au Cameroun.

**Méthodes:**

il s´est agi d´une étude rétrospective, pendant la période du 1^er^ janvier 2016 au 31 décembre 2017.

**Résultats:**

trois-cent-cinquante-sept (357) femmes ont été incluses. L´âge variait de 25 à 88 ans avec une moyenne de 52,82 ± 12,36 ans. Les patientes originaires de la région de l´Ouest étaient les plus représentées avec un pourcentage de 42,2% (n= 124/294). Il s´agissait majoritairement de ménagères, sans emploi avec une fréquence de 57,3% (n=200/341). L´âge au premier rapport sexuel était renseigné chez seulement 37% (n=133/357) de la population, avec une moyenne de 16,73 ± 2,16 ans; tandis que la moyenne d´âge au premier accouchement était de 18,92 ± 3,44 ans. Par ailleurs, 6,5% (n= 11/169) de la population est tabagique tandis que 44 % (n=73/166) est alcoolique. Le carcinome épidermoïde était le type histologique le plus fréquent (85,6%, n=255/298). Les stades les plus fréquents au diagnostic étaient les stades IIB (22,3%, n=71/319) suivis des stades IIIB (21,6%, n=69/319).

**Conclusion:**

le cancer du col de l´utérus au Cameroun est une pathologie de la femme adulte, sans emploi, avec des stades avancés au diagnostic. D´où la nécessité d´améliorer la sensibilisation sur la prévention et le diagnostic précoce des cas.

## Abstract (en)

**Introduction:**

cervical cancer is a public health problem in Cameroon, ranking as the 2^nd^ most frequent cancer. The purpose of our study was to describe the epidemiological and clinical characteristics of patients with cervical cancer at the Douala General Hospital in Cameroon.

**Method:**

we conducted a retrospective study over the period 1 January 2016 to 31 December 2017.

**Results:**

the study enrolled 357 women. Patients´ age ranged from 25 to 88 years, with an average age of 52.82 ± 12.36 years. Patients from the Western Region were more heavily represented, with a percentage of 42.2% (n= 124/294). The majority of them were unemployed housekeepers (57.3%; n=200/341). The age of first sexual intercourse was recorded for only 37% (n=133/357) of the study population, with an average age of 16.73 ± 2.16 years; while the average age on giving birth to the first child was 18.92 ± 3.44 years. On the other hand, 6.5% (n=11/169) of patients were smokers, while 44% (n=73/166) were alcohol abusers. Squamous cell carcinoma was the most common histologic type (85.6%; n=255/298). The most frequent stage at diagnosis was stage IIB (22.3%, n=71/319) followed by stage IIIB (21.6%; n=69/319).

**Conclusion:**

in Cameroon, cervical cancer commonly occurs in unemployed adult women and it is associated with an advanced-stage diagnosis. Hence the need to improve awareness of prevention and early diagnosis.

## Introduction

Le cancer du col de l´utérus est le 4^e^ cancer de la femme dans le monde, avec une incidence de 604 127 nouveaux cas en 2020 [1,2]. L´amélioration des moyens de dépistage d´une part, l´augmentation de l´espérance de vie, la croissance démographique, l´urbanisation, et le changement de modes de vie d´autre part ont contribué à une augmentation de plus de 50 % de l´incidence des cancers entre 2008 et 2018 [1-3]. Dans le même sens, l´amélioration de la connaissance des facteurs de risque et de nouvelles techniques thérapeutiques ont donné lieu à la mise en route de politiques mondiales de prévention, de dépistage et de prise en charge précoce de cette affection [4,5].

En Afrique, le cancer du col de l´utérus occupe le deuxième rang en termes d´incidence et de mortalité avec 117 316 nouveaux cas et 76 745 décès en 2020 [1,2]. Au Cameroun, le registre de cancer n´est pas opérationnel depuis 2012. Selon les données de GLOBOCAN, le cancer du col occuperait le 2^e^ rang des cancers en termes d´incidence et de mortalité tous sexes confondus, avec 2770 nouveaux cas et 1 787 décès [1,2]. Quelques auteurs ont décrit des caractéristiques de cette maladie. C´est le cas de Sando *et al*., qui décrivent 49,5% (n=210) de cancers du col dans la région du Centre du Cameroun, avec majoritairement des stades II au diagnostic (37%, n=100/270) [6,7]. Nkufsai *et al*. quant à eux ont décrivent dans la région du Nord-Ouest, 52% (n=31) de cancers du col, chez les patientes de 50 à 54 ans, avec des stades avancés (47,7% de stades 3 et 4) [6,7]. Enow-Orock *et al*., dans la région du Centre, retrouvaient 40,18% de cancer du col [8].

Nous avons mené une étude sur le profil de cette maladie au Service de Radiothérapie de l´Hôpital Général de Douala, qui est l´unique centre de radiothérapie public fonctionnel dans le pays et qui reçoit les patients de toutes les régions. Cette étude visait à mieux orienter les politiques en matière de lutte contre cette maladie. Pour ce faire, notre objectif était de décrire les caractéristiques épidémiologiques et cliniques des patientes porteuses d´un cancer du col de l´utérus à l´Hôpital Général de Douala.

## Méthodes

**Conception et cadre de l´étude:** nous avons mené une étude hospitalière, descriptive, rétrospective pendant la période du 1^er^ janvier 2016 au 31 décembre 2017, au Service de Radiothérapie de l´Hôpital Général de Douala.

**Population de l´étude:** la population cible était les patientes atteintes de cancer du col de l´utérus au Cameroun. La population source était constituée des patientes suivies pour cancer du col de l´utérus au service de radiothérapie de l´Hôpital Général de Douala. Etaient inclus dans notre étude les femmes, de tout âge, porteuses d´un cancer du col de l´utérus, confirmé à l´examen anatomopathologique, tous stades confondus, suivies au service de Radiothérapie de l´Hôpital Général de Douala. Ce service est l´unique service public fonctionnel au Cameroun depuis 2012. Nous avons exclu les patientes dont les dossiers étaient incomplets et ne permettaient pas une exploitation des données qui s´y trouvaient. Ces dossiers étaient considérés comme inexploitables. La technique d´échantillonnage utilisée était non probabiliste, basée sur le recrutement consécutif et exhaustif de toutes les patientes obéissant aux critères d´inclusion.

**Collecte des données:** elle s´est effectuée à l´aide des dossiers des patients conservés dans le service des archives de radiothérapie, les registres de consultation et les fiches de traitement des malades. Les données ont été consignées dans une fiche de collecte standardisée, pré-testée. Les données épidémiologiques recherchées étaient l´âge de la patiente, le lieu de résidence, la région d´origine, la profession, l´âge au premier rapport sexuel, l´âge au premier accouchement, la consommation de tabac et d´alcool. Quant aux données cliniques, il s´agissait de l´état général au diagnostic, du type histologique et du stade au diagnostic selon la classification de la Fédération Internationale de Gynécologie et d´Obstétrique (FIGO).

La Fédération Internationale de Gynécologie et d´Obstétrique (FIGO) classe les stades de cancer du col de l´utérus qu´il suit [9]: stade IA: cancer invasif identifié par examen microscopique uniquement. L´invasion est limitée à l´invasion stromale mesurée ne dépassant pas 5mm en profondeur et 7 mm en largeur. Stade IA1: l´invasion mesurée dans le stroma ne dépasse pas 3 mm en profondeur et 7 mm en largeur. Stade IA2: l´invasion mesurée dans le stroma est comprise entre 3 et 5 mm en profondeur et ne dépasse pas 7 mm en largeur. Stade IB: soit les lésions cliniques sont limitées au col, soit les lésions infracliniques sont plus importantes que dans le Stade IA. Toute lésion macroscopiquement visible même avec une invasion superficielle est classée cancer de Stade IB. Stade IB1: lésions cliniques de taille ne dépassant pas 4 cm. Stade IB2: lésions cliniques de taille supérieure à 4 cm. Stade II: tumeur s´étendant au-delà de l´utérus mais ne touchant pas la paroi pelvienne ni le tiers inférieur du vagin. Stade IIA: pas d´atteinte paramétriale évidente. L´invasion touche les deux tiers supérieurs du vagin. Stade IIB: atteinte paramétriale évidente, mais la paroi pelvienne n´est pas touchée. Stade III: tumeur s´étendant jusqu´à la paroi pelvienne et/ou atteignant le tiers inférieur du vagin et/ou avec hydronéphrose ou rein non fonctionnel. Stade IIIA: tumeur atteignant le tiers inférieur du vagin sans extension à la paroi pelvienne. Stade IIIB: tumeur s´étendant jusqu´à la paroi pelvienne et/ou avec hydronéphrose ou rein non fonctionnel. Stade IV: extension aux organes pelviens ou à distance. Stade IVA: atteinte de la muqueuse rectale ou vésicale. Stade IVB: extension au-delà du pelvis. Nous nous sommes servis de toutes les informations disponibles dans les dossiers médicaux, les fiches de traitement de radiothérapie et les rapports de résultats d´examen disponibles dans les dossiers pour éviter au maximum les biais d´information.

**Analyse statistique:** nous avons analysé les données grâce au logiciel Epi info 3.5.3 et Microsoft Office Excel 2010. Les données ont été présentées sous forme de tableaux et de figures. Les statistiques descriptives ont été appliquées pour condenser et ordonner les variables. Les variables qualitatives ont été exprimées par leur représentation graphique au travers de diagrammes en bâton ou en secteur. Les variables quantitatives ont été exprimées par la moyenne accompagnée de l´écart-type.

**Considérations éthiques:** nous avons obtenu l´autorisation de l´Hôpital Général de Douala. L´étude étant rétrospective, les données ont été obtenus au travers des dossiers des patientes. La confidentialité de toutes les informations a été assurée.

## Résultats

Entre le 1^er^ janvier 2016 et le 31 décembre 2017, 357 femmes ont été consultées pour un cancer du col de l´utérus au service de radiothérapie de l´Hôpital Général de Douala. Toutes ces patientes répondaient aux critères d´inclusion et ont été toutes incluses dans l´étude.

### Caractéristiques socio-épidémiologiques

**Figure 1:**
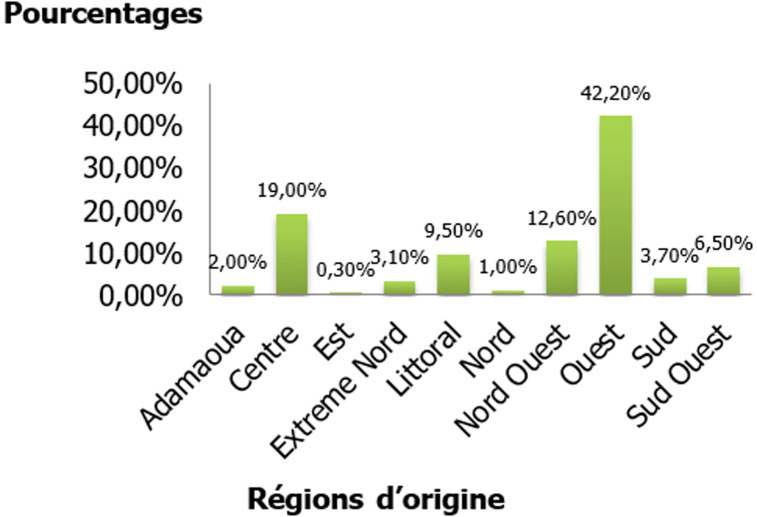
distribution des patientes selon la région d´origine

**Figure 2:**
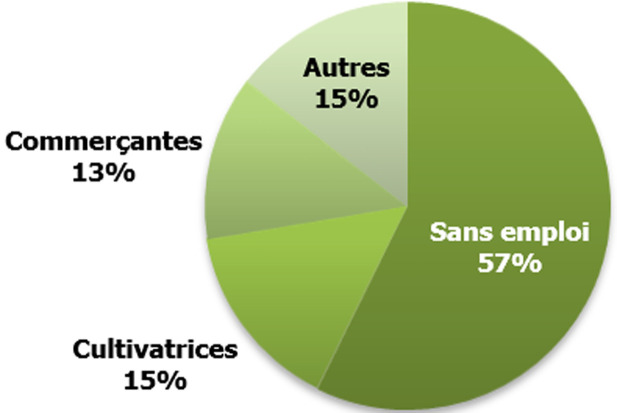
distribution des patientes selon leur profession

L´âge des patientes variait de 25 à 88 ans avec une moyenne de 52,82 ± 12,36 ans. La tranche d´âge la plus représentée était celle de 45 à 54 ans (Tableau 1). Parmi ces patientes, 16 (4,5%) résidaient hors du pays et n´avaient effectué leur déplacement que pour leur traitement. Les 341 patientes restantes quant à elles résidaient au Cameroun et provenaient des différentes régions du pays. Parmi ces dernières, 26,3% (n=90) résidaient à Douala, 18% (n=61) à Yaoundé et 6% (n= 20) à Bamenda. Les régions d´origine étaient connues chez 294 patientes, soit 82,35% de la population. Les patientes originaires de la région de l´Ouest étaient les plus représentées avec un pourcentage de 42,2% (n= 124/294), suivie de celles de la région du Centre (19%, n=56), du Nord-Ouest (12,6%, n=37) et du Littoral (9,5%, 28) (Figure 1). Parmi les 349 patientes ayant renseigné leur profession, la majorité était des femmes au foyer, sans emploi (57,3%, n=200), puis suivaient les cultivatrices (15,1%, n=53), les commerçantes (13,1%, n= 46) et divers autres corps de métiers (Figure 2). L´âge au premier rapport sexuel était renseigné chez seulement 37% (n= 133) de la population. La moyenne de cet âge était de 16,73 ± 2,16 ans avec des extrêmes de 12 ans et 22 ans. Dans la même lancée la moyenne d´âge au premier accouchement dans cette population était de 18,92 ± 3,44 ans avec des extrêmes de 13 ans et 36 ans (n=153) (Tableau 2). La consommation de tabac et d´alcool a été rapportée par 6,5% (n=11/169) et 44% (n=73/166) des patientes respectivement (Tableau 2).

**Tableau 1:** répartition des patientes par tranches d´âge

Tranches d´âge	Effectifs (n)	Fréquences (%)
[25-35[	24	6,7
[35-45[	78	21,8
[45-55[	100	28,0
[55-65[	88	24,6
[65-75[	49	13,7
[75-85[	16	4,5
[85-95[	2	0,6
Total	357	100

**Tableau 2:** caractéristiques épidémiologiques et cliniques des patientes

Variables	Effectifs (n)	Fréquences (%)
**Age au 1^er^ rapport sexuel**	133	37,25
	Moyenne: 16,73 ± 2,16 ans	
**Age au 1^er^ accouchement**	153	42,85
	Moyenne: 18,92 ± 3,44 ans	
**Consommation de tabac**	169	47,33
**Présence**	11	6,5
**Absence**	169	93,5
**Consommation d´alcool**	166	46,49
**Présence**	73	44
**Absence**	93	56
**Etat général OMS**	324	90,75
**OMS 1**	316	97,5
**≥ OMS 2**	8	2,5
**Type histologique**	298	83,47
**Carcinome épidermoïde**	255	85,6
**Adénocarcinome**	23	7,7
**Autres**	20	6,7
**Stades au diagnostic**	319	89,35
**IA**	1	0,30
**IA2**	8	2,50
**IB**	1	0,30
**IB1**	18	5,60
**IB2**	27	8,50
**IIA**	44	13,80
**IIB**	71	22,30
**IIIA**	21	6,60
**IIIB**	69	21,60
**IVA**	37	11,60
**IVB**	22	6,90

### Caractéristiques cliniques

La majorité des femmes avait un bon état général à la première consultation (97,5%, n= 316/324). Le carcinome épidermoïde était le type histologique le plus fréquent (85,6%, n= 255/298) puis quelques cas d´adénocarcinome (7,7%, n= 23/298) et d´autres types histologiques (6,7%, n=20/298). Parmi ces patientes, 69% (n=220/319) avaient des stades avancés au diagnostic (IIB et +). Les stades les plus fréquents au diagnostic étaient les stades IIB (22,3%, n=71/319) suivis des stades IIIB (21,6%, n=69/319) (Tableau 2).

## Discussion

Notre travail portait sur la description du profil épidémiologique et clinique des patientes suivies pour cancer du col de l´utérus au service de radiothérapie de l´Hôpital Général de Douala. L´âge des patientes variait entre 22 et 88 ans, avec une moyenne de 52,82 ± 12,36 ans. Il s´agissait majoritairement de femmes au foyer, sans emploi (57,3%). L´âge au premier rapport sexuel était renseigné chez seulement 37% de la population avec une moyenne de 16,73 ± 2,16 ans; tandis que la moyenne d´âge au premier accouchement était de 18,92 ± 3,44 ans. Par ailleurs, 6,5% de la population était tabagique tandis que 44 % était alcoolique. La majorité des femmes avait un bon état général à la première consultation (97,5%). Le carcinome épidermoïde était le type histologique le plus fréquent (85,6%) puis quelques cas d´adénocarcinome (7,7%) et d´autres types histologiques (6,7%). Parmi ces patientes, 69% avaient des stades avancés au diagnostic (IIB et +). Les stades les plus fréquents au diagnostic étaient les stades IIB (22,3%) suivis des stades IIIB (21,6%).

L´âge au diagnostic retrouvé dans notre population se rapproche de celui de quelques études africaines. En effet, l´âge moyen au diagnostic de cancer du col de l´utérus était de 52,43 ± 3,82 ans dans une étude camerounaise menée en 2013 sur le profil des cancers gynécologiques et mammaires à Yaoundé au Cameroun; et une autre en 2019 à Bamenda au Cameroun retrouvait des résultats similaires [6,7]. Enow Orock *et al*., retrouvaient un âge moyen de 51,65 ans et une médiane d´âge de 47,2 ans dans leur étude sur le profil des cancers gynécologiques à Yaoundé au Cameroun [8]. Des résultats similaires ont été observés au Maroc dans leur cohorte de patientes suivies pour cancer du col de l´utérus de 2003 à 2007, qui retrouvait un âge moyen au diagnostic de 52,1 ± 11,8 ans [10]. La Tanzanie elle aussi enregistrait une population avec une moyenne d´âge de 48,8 ans au diagnostic de cancer du col de l´utérus [11]. En revanche, en Chine, une population plus jeune au diagnostic de cancer du col a été observée. L´âge médian au diagnostic était de 42 ans [12].

Dans notre étude, 57,3% des patientes étaient femmes au foyer, sans emploi. Des résultats similaires ont été décrits par Enow Orock *et al*. qui retrouvaient dans leur cohorte de 193 femmes, 44.56% de femmes au foyer, sans emploi, 12.43%, de personnels de bureau et 30.05% de cultivatrices chez les patientes avec carcinome épidermoïde; 58.33% de femmes au foyer, sans emploi, 33.33% de personnels de bureau et 8.33% de cultivatrices chez celles avec un adénocarcinome [8]. Le Maroc présente une plus grande proportion de patientes sans emploi, avec 90,7% de femmes au foyer sans emploi dans leur cohorte de 890 femmes suivies pour cancer du col entre 2003 et 2007 [10].

Dans notre étude, la moyenne d´âge au premier rapport sexuel était 16,73 ± 2,16 ans. Ces résultats se rapprochent de ceux de Tebeu *et al*. en 2005, qui retrouvaient une moyenne d´âge au premier rapport sexuel de 19,4 ans et 17,9 ans respectivement dans la population générale et chez les patientes dans leur étude sur les lésions précancéreuses du col utérin en zone rurale au Cameroun [13].

Dans notre série, 69% des patients avaient des stades avancés au diagnostic (IIB et +). Les stades avancés au diagnostic, plus observés dans notre étude ont également été rapportés dans plusieurs autres études. D´abord au Cameroun, Nkfusai *et al*. le décrivent en 2019, dans leur cohorte de femmes suivies pour cancer du col, où 47,5% avaient des stades avancés (III et IV au diagnostic) [7].

Comme le décrit les données de la littérature, dans notre étude le carcinome épidermoïde était le type histologique le plus fréquent (85,6%) puis quelques cas d´adénocarcinome (7,7%) et d´autres types histologiques (6,7%). Enow Orock *et al*. retrouvaient 87,33% de carcinomes épidermoïdes, 5,43% d´adénocarcinome et 1,8% de carcinome adénosquameux [8]. Résultats similaires à ceux de Sando *et al*. en 2014, avec 87,6% de carcinomes épidermoïdes [6]. Berraho *et al*. au Maroc retrouvaient plus de 90,5% de carcinomes épidermoïdes dans leur population entre 2003 et 2007, 5% d´adénocarcinomes et 4,5% d´autres types histologiques, tout comme les données recueillies en Tunisie et en Algérie [10,14].

Kidanto *et al*. en Tanzanie quant à eux retrouvaient plus de 90% des stades avancés au diagnostic (IIB-IV) [11]. Berraho *et al*. au Maroc décrivaient 82,8% de stades avancés (II-IV), avec 43,7% de stades II, 31,8% de stades III et 6,3% de stades IV. Données similaires en Tunisie et en Algérie [10,14]. Par contre Sando *et al*., en 2014 avaient 62.9% de stades I et II au diagnostic dans leur série [6].

Bien que nous ayons souhaité décrire le profil épidémiologique et clinique des patientes porteuses de cancer du col de l´utérus à l´Hôpital Général de Douala, au Cameroun, cette étude pourrait comporter quelques limites. La première est un biais de sélection, dû au fait que cette étude est réalisée dans l´unique centre de radiothérapie public du pays et donc exclut les patientes qui n´ont pas souhaité ou pu réaliser une radiothérapie. Par ailleurs, cette étude ne tient pas compte des patientes qui auraient pu bénéficier d´une radiothérapie dans un centre privé disponible au Cameroun. En outre, étant donné le caractère rétrospectif de cette étude, nous pouvons faire face à un biais d´informations car l´étude se limite aux informations disponibles dans le dossier médical des patientes. Tout ceci pourrait rendre difficile la généralisation de nos résultats. Nous nous sommes servis de toutes les informations disponibles dans les dossiers médicaux, les fiches de traitement de radiothérapie et les rapports de résultats d´examen disponibles dans les dossiers pour éviter au maximum ces biais d´information. Toutefois le service de radiothérapie de l´Hôpital Général de Douala étant le seul service public fonctionnel du pays, ces données sont un reflet en matière de profil des patientes suivies pour cancer du col de l´utérus et qui ont bénéficié d´une radiothérapie.

## Conclusion

Le cancer du col utérin touche des patientes adultes, majoritairement originaire de la région de l´Ouest Cameroun et sans emploi. Près de la moitié des patientes est alcoolique. L´activité sexuelle est peu décrite. Les patientes atteintes de cette maladie étaient reçues à des stades avancés de la maladie. Ces résultats constituent une base de décision pour le développement de stratégies pour l´amélioration de la lutte contre le cancer du col au Cameroun. Ils mettent l´emphase sur la nécessité d´améliorer la sensibilisation sur les facteurs de risque, sur la vaccination contre le Virus du Papillome Humain, sur le dépistage et le diagnostic précoce de cette maladie dans notre pays.

### Etat des connaissances sur le sujet

- Le cancer du col est un problème de santé publique au Cameroun;
- Les stades au diagnostic de cancer du col de l´utérus au Cameroun sont tardifs;
- Le carcinome épidermoïde est le type histologique le plus atteint.

### Contribution de notre étude à la connaissance

- La tranche d´âge la plus touchée par cette affection varie entre 45 à 54 ans;
- Une consommation d´alcool et de tabac, est observée respectivement chez 44% et 6,5% de patientes atteintes de cancer du col;
- La moyenne d´âge au premier rapport sexuel était de 16,73 ± 2,16 ans avec des extrêmes de 12 ans et 22 ans.

## References

[1] Sung H, Ferlay J, Siegel RL, Laversanne M, Soerjomataram I, Jemal A (2021). Global Cancer Statistics 2020: GLOBOCAN Estimates of Incidence and Mortality Worldwide for 36 Cancers in 185 Countries. CA Cancer J Clin.

[2] Ferlay J, Colombet M, Soerjomataram I, Mathers C, Parkin DM, Piñeros M (2019). Estimating the global cancer incidence and mortality in 2018: GLOBOCAN sources and methods. Vol. 144. International Journal of Cancer.

[3] Ferlay J, Shin HR, Bray F, Forman D, Mathers C, Parkin DM (2010). Estimates of worldwide burden of cancer in 2008: GLOBOCAN 2008. Int J Cancer.

[4] Bhatla N, Aoki D, Sharma DN, Sankaranarayanan R (2018). Cancer of the cervix uteri. Int J Gynecol Obstet.

[5] OMS (2005). La lutte contre le cancer du col de l´utérus Guide des pratiques essentielles Deuxième édition Pour plus d´informations. Équipe OMS de coordination.

[6] Sando Z, Tsuala Fouogue J, Ymele Fouelifack F, Hortence Fouedjio J, Telesphore Mboudou E, Oyono Essame JL (2014). Profil des cancers gynécologiques et mammaires à Yaoundé-Cameroun. Pan Afr Med J.

[7] Nkfusai NC, Cumber SN, Williams T, Kimbi JKA, Yankam BM, Anye CS (2019). Cervical cancer in the Bamenda Regional Hospital, North West Region of Cameroon: A retrospective study. Pan Afr Med J.

[8] Enow-Orock G, Mbu R, Ngowe N, Tabung F, Mboudou E, Ndom P (2006). Gynecological cancer profile in the Yaounde population, Cameroon. Clin Mother Child Health.

[9] Mahjoub Marzouk F, Jalaguier-Coudray A (2021). Rim Villard-Cancer du col utérin: nouvelle classification de la Fédération internationale de gynécologie et d´obstétrique. Imagerie de la Femme.

[10] Berraho M, Bendahhou K, Obtel M, Zidouh A, Benider A, Errihani H (2012). Cervical cancer in morocco: Epidemiological profile from two main oncological centers. Asian Pac J Cancer Prev.

[11] Kidanto HL, Kilewo CD, Moshiro C (2002). Cancer of the cervix: Knowledge and attitudes of female patients admitted at Muhimbili National Hospital, Dar es Salaam. East Afr Med J.

[12] Shu P, Li R, Xie D, He Y, Wang X, Li Q (2020). Clinical profile and treatment outcome of collision carcinoma in cervix. Medicine (Baltimore).

[13] Tebeu PM, Sandjong I, Nkele N, Fokoua S, Achu P, Kouam L (2005). Lésions pré-cencereuses du col uterin en zone rurale: étude transversale. Médecine d´Afrique Noire.

[14] Yazghich I, Berraho M (2018). cancer in the maghreb country (Morocco-Algeria-Tunisia): Epidemiological, clinical profile and control policy. Tunisie Medicale. Maison du Medicine.

